# Outcomes and Risk Score for Distal Pancreatectomy with Celiac Axis Resection (DP-CAR): An International Multicenter Analysis

**DOI:** 10.1245/s10434-018-07101-0

**Published:** 2019-01-04

**Authors:** Sjors Klompmaker, Niek A. Peters, Jony van Hilst, Claudio Bassi, Ugo Boggi, Olivier R. Busch, Willem Niesen, Thomas M. Van Gulik, Ammar A. Javed, Jorg Kleeff, Manabu Kawai, Mickael Lesurtel, Carlo Lombardo, A. James Moser, Ken-ichi Okada, Irinel Popescu, Raj Prasad, Roberto Salvia, Alain Sauvanet, Christian Sturesson, Matthew J. Weiss, Herbert J. Zeh, Amer H. Zureikat, Hiroki Yamaue, Christopher L. Wolfgang, Melissa E. Hogg, Marc G. Besselink, the E-AHPBA DP-CAR study group, Sarah L. Gerritsen, Mustapha Adham, M. Teresa Albiol Quer, Frederik Berrevoet, Manuela Cesaretti, Raffaele Dalla Valle, Benjamin Darnis, Markus K. Diener, Marco Del Chiaro, Thilo H. Hackert, Robert Grützmann, Traian Dumitrascu, Helmut Friess, Seiko Hirono, Arpad Ivanecz, Anastasios Karayiannakis, Giuseppe K. Fusai, Knut J. Labori, Santiago López-Ben, Jean-Yves Mabrut, Motoki Miyazawa, Willem Niesen, Fernando Pardo, Julie Perinel, Geert Roeyen

**Affiliations:** 1 Department of Surgery, Cancer Center Amsterdam, Amsterdam UMC, University of Amsterdam, Amsterdam, The Netherlands; 2 Department of Surgery, Johns Hopkins Hospital, Baltimore, MD USA; 3 Department of Surgery, University of Utrecht Medical Center, Utrecht, The Netherlands; 4 Department of Surgery, Pancreas Institute University of Verona, Verona, Italy; 5 Division of General and Transplant Surgery, University of Pisa, Pisa, Italy; 6 Department of General, Visceral and Transplantation Surgery, Heidelberg University, Heidelberg, Germany; 7 Department of Visceral, Vascular and Endocrine Surgery, Martin-Luther-University Halle-Wittenberg, Halle, Saale, Germany; 8 Second Department of Surgery, Wakayama Medical University, Wakayama, Japan; 9 Department of Surgery and Liver Transplantation, Croix-Rousse University Hospital, Hospices Civils de Lyon, University of Lyon I, Lyon, France; 10 The Pancreas and Liver Institute, Beth Israel Deaconess Medical Center, Harvard Medical School, Boston, MA USA; 11 Center of General Surgery and Liver Transplant, Fundeni Clinical Institute, Bucharest, Romania; 12 Department of HPB and Transplant Services, National Health Service, Leeds, UK; 13 Department of HPB Surgery, Hôpital Beaujon, APHP, University Paris VII, Clichy, France; 14 Division of Surgery, Department for Clinical Science, Intervention and Technology (CLINTEC), Karolinska Institutet at Karolinska University Hospital, Stockholm, Sweden; 15 Department of Surgery, University of Texas Southwestern Medical Center, Dallas, TX USA; 16 Department of Surgery, University of Pittsburgh Medical Center, Pittsburgh, PA USA; 17 Department of Surgery, Northshore University HealthSystem, Chicago, IL USA

## Abstract

**Background:**

Distal pancreatectomy with celiac axis resection (DP-CAR) is a treatment option for selected patients with pancreatic cancer involving the celiac axis. A recent multicenter European study reported a 90-day mortality rate of 16%, highlighting the importance of patient selection. The authors constructed a risk score to predict 90-day mortality and assessed oncologic outcomes.

**Methods:**

This multicenter retrospective cohort study investigated patients undergoing DP-CAR at 20 European centers from 12 countries (model design 2000–2016) and three very-high-volume international centers in the United States and Japan (model validation 2004–2017). The area under receiver operator curve (AUC) and calibration plots were used for validation of the 90-day mortality risk model. Secondary outcomes included resection margin status, adjuvant therapy, and survival.

**Results:**

For 191 DP-CAR patients, the 90-day mortality rate was 5.5% (95 confidence interval [CI], 2.2–11%) at 5 high-volume (≥ 1 DP-CAR/year) and 18% (95 CI, 9–30%) at 18 low-volume DP-CAR centers (*P *= 0.015). A risk score with age, sex, body mass index (BMI), American Society of Anesthesiologists (ASA) score, multivisceral resection, open versus minimally invasive surgery, and low- versus high-volume center performed well in both the design and validation cohorts (AUC, 0.79 vs 0.74; *P* = 0.642). For 174 patients with pancreatic ductal adenocarcinoma, the R0 resection rate was 60%, neoadjuvant and adjuvant therapies were applied for respectively 69% and 67% of the patients, and the median overall survival period was 19 months (95 CI, 15–25 months).

**Conclusions:**

When performed for selected patients at high-volume centers, DP-CAR is associated with acceptable 90-day mortality and overall survival. The authors propose a 90-day mortality risk score to improve patient selection and outcomes, with DP-CAR volume as the dominant predictor.

**Electronic supplementary material:**

The online version of this article (10.1245/s10434-018-07101-0) contains supplementary material, which is available to authorized users.

Distal pancreatectomy with celiac axis resection (DP-CAR) may lead to a radical resection for selected patients with locally advanced pancreatic cancer involving the celiac axis. The procedure relies on collateral flow from the superior mesenteric artery via the pancreatic head arcade to the liver and stomach. Some centers perform preoperative embolization of the hepatic and/or left gastric artery^1^ to maximize the formation of collaterals and reduce the risk of ischemia-related complications, but evidence in support of this practice is lacking.

A recent systematic review of 250 patients,^2^ four subsequent single-center studies of 17–80 patients,^3^–^6^ and a pan-European retrospective multicenter study of 68 patients^7^ all suggested that DP-CAR for pancreatic ductal adenocarcinoma leads to an acceptable overall survival ranging from 17 to 35 months. Despite this survival benefit, the 90-day mortality rate after DP-CAR can be as high as 16–18%.^7^–^9^ A clinical risk score that evaluates the risk of mortality before surgery could inform shared decision making and improve outcomes through better patient selection. However, such a score is difficult to design for low-volume procedures such as DP-CAR.

A recent study and subsequent international validation (Klompmaker et al., unpublished data) proposed a risk prediction model for major morbidity (including mortality) after standard distal pancreatectomy based on age, sex, American Society of Anesthesiology (ASA) score, body mass index (BMI), multivisceral resection, and open versus minimally invasive approach.^10^ This model reflects the general risk for severe adverse events compared with the risk for specific complications after distal pancreatectomy. Although DP-CAR is a more extensive procedure, the existing model could serve as a basis for a clinical risk score. It is known that this method of adjustment is superior to designing of a new model with many fewer patients.^11^

This study aimed to create a 90-day mortality risk score for an existing cohort from 20 European DP-CAR centers and to perform an international validation for a combined cohort derived from three very-high-volume centers in the United States and Japan. We hypothesized that the risk score could successfully identify high-risk patients with little expected benefit from DP-CAR.

## Methods

For this multicenter retrospective study, we used a design cohort, previously collected at 20 European high-volume pancreas surgery centers,^7^ and a retrospective multicenter validation cohort, which included patients from Johns Hopkins Hospital (JHH), Baltimore, MD, USA (2004–2017), the University of Pittsburgh Medical Center (UPMC), Pittsburgh, PA, USA (2007–2017), and the Wakayama Medical University Hospital (WMUH), Wakayama, Japan (2004–2017).

We added and updated the previously published series^3^,^4^,^12^ comprising the validation cohort. We used the design cohort to create a 90-day mortality risk score based on the coefficients of a previously validated prediction model for major morbidity, described later in more detail.

The study was designed according to the Strengthening the Reporting of Observational Studies in Epidemiology (STROBE) guidelines for observational studies.^13^ A predefined study protocol, including methods and authorship agreements, was distributed among the participating centers. The need for ethical approval was waived by the institutional review board at the Academic Medical Center in Amsterdam.

### Exposures and Outcomes

The DP-CAR procedure was performed in four main variations: (1) standard DP-CAR with resection of the common hepatic, the left gastric, and the splenic arteries,^14^ (2) DP-CAR with superior mesenteric or portal vein resection, (3) DP-CAR with hepatic artery reconstruction (for insufficient flow), and (4) DP-CAR with left gastric artery preservation or bypass reconstruction.^12^ In this study, all variations were treated equally, but the associations between the type of DP-CAR and 90-day mortality were assessed. The primary outcome was 90-day mortality. The secondary outcomes were major morbidity (Clavien-Dindo 3a–4b), resection margin status, adjuvant therapy, and survival.

### Data Extraction and Definitions

In both the design and validation cohorts, data were extracted without using patient identifiers. For the validation cohort, we queried all three local databases for patients who underwent DP-CAR for all indications (premalignant or malignant) in adult patients (≥ 18). We updated survival on existing cases and added new cases until 1 June 2017.

The preoperative variables included baseline characteristics (age, sex, BMI, Charlson comorbidity index, surgical history), performance status (ASA classification), vascular and/or organ involvement, and tumor etiology. Procedures were considered multivisceral if additional organ resections besides those for pancreas, gallbladder, or spleen were performed.

The annual DP-CAR case volume per center was based on the average number of reported procedures between 1 January 2014 and 31 December 2016. Centers with an annual case volume of one or more were considered high-volume DP-CAR centers, and others were considered low-volume DP-CAR centers. Due to the lack of variability, annual volume could not be used as a continuous variable.

Postoperative complications were scored and classified according to the Clavien-Dindo classification.^15^ International Study Group on Pancreatic Surgery (ISGPS) definitions were used to classify delayed gastric emptying, post-pancreatectomy hemorrhage, and pancreatic fistulas.^16^–^18^ Postoperative organ space infections were defined according to the Center for Disease Control and Prevention (CDC) definition.^19^

Centers were asked to categorize resection margins according to the Royal College of Pathologists^20^ definitions as follows: R0 (distance from margin to tumor ≥ 1 mm), R1 (distance from margin to tumor < 1 mm), and R2 (macroscopically positive margin). All complications were recorded at the index hospitalization and at subsequent readmissions up to 90 days. Survival was based on the last recorded moment of contact between a patient and a hospital staff member.

### Statistical Analysis

We used predictor estimates from an internationally designed (*n* = 1661) prediction model for major morbidity (including mortality) after distal pancreatectomy (without arterial resection) based on age, sex, ASA, BMI, multivisceral resection, and open versus minimally invasive approach (Klompmaker et al., unpublished data). We created a risk score by rounding and multiplication of the original model (beta) coefficients.

We tested the model with the design cohort using only baseline (intercept) adjustment and performed validation according to the recommendations by Moons et al.^11^ Validation included addition of new prediction factors based on a univariate screen and forward stepwise selection (*P* < 0.1). Both the intercept adjustment and new predictor coefficients were calculated using the method described by Janssen et al.^21^ We determined the model’s ability to identify high- versus low-risk patients (discrimination) by comparing the area under the receiver operating curve (AUC)^22^ in both cohorts using the DeLong test.^23^ We assessed the accuracy of the risk model predictions per risk quantile using calibration plots.^24^

Categorical variables are reported as counts and proportions and continuous variables as means ± standard deviations and/or as medians (interquartile ranges) based on normality. To determine statistical significance (alpha 0.05), we used Fisher’s exact test for categorical variables and the Mann–Whitney *U* test for continuous variables. Kaplan–Meier estimation was used to assess overall survival. All confidence intervals are 95%. The data were analyzed using STATA version 15.0 (StataCorp LP, College Station, TX, USA).

## Results

**Fig. 1:**
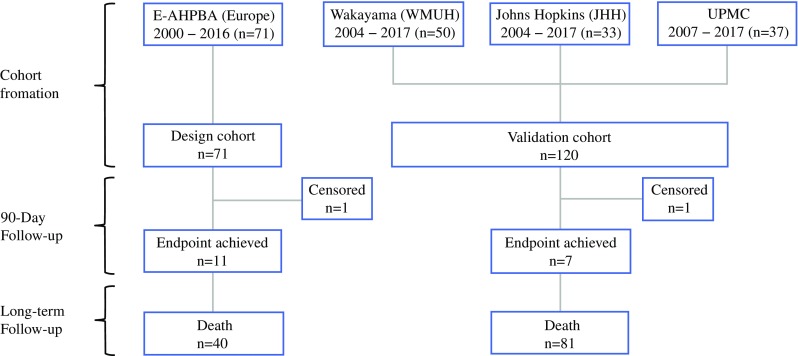
Study flow chart of data sources and year of inclusion. Two cases were lost to follow-up evaluation within 90 days after surgery. E-AHPBA, European-African Hepato-Pancreato-Biliary Association; JHH, Johns Hopkins Hospital; UPMC, University of Pittsburgh Medical Center; WMUH, Wakayama Medical University Hospital

The study investigated 191 patients undergoing DP-CAR between 1 January 2000 and 31 June 2017. The design cohort contained 71 patients, and the validation cohort comprised 120 patients. Of these patients, 33 were treated at JHH, 37 at UPMC, and 50 at WMUH. The median follow-up period was 309 days for the design cohort and 447 days for the validation cohort. Overall, 90-day mortality occurred for 18 (9.5%) of the 191 patients. These 18 patients included 11 (16%) in the design cohort and 7 (5.8%) in the validation cohort (Fig. 1).

The recorded causes of mortality were gastric ischemia (*n *= 4), post-pancreatectomy hemorrhage (*n* = 4), pneumonia (*n* = 3), liver ischemia (*n* = 2), abdominal infection (*n* = 2), sepsis with multi-organ failure (*n* = 2), and progression of residual cancer (*n* = 1). The additional 90-day major morbidity (Clavien-Dindo 3a–4b) rate was 27%, which was comparable in the two cohorts. For 174 patients with pancreatic ductal adenocarcinoma, the R0-resection rate was 60% (*n* = 113), and the median overall survival period was 19 months (95% CI 15–25 months). The survival rate did not differ between the patients with R0 and R1 resections.

### Baseline

The baseline characteristics are presented in Table 1. The differences between populations based on the geographic region of origin (Europe vs WMUH vs JHH/UPMC) are presented in Supplements 1A and 1B. At baseline, the patients from WMUH were less often female (Europe vs WMUH vs JHH/UPMC: 48% vs 36% vs 50%, respectively), were older (median 60 vs 68 vs 62 years), and had a lower BMI (mean 24 vs 22 vs 26) than the European and JHH/UPMC-series. Patients at JHH/UPMC were more likely to have an ASA of 3 or 4 (8% vs 19% vs 84%) and more likely to receive neoadjuvant therapy (51% vs 52% vs 94%). Hepatic and/or gastric artery embolization was routinely performed at WMUH (92%), and in some cases in Europe (23%), but never at JHH/UPMC (0%). The DP-CAR procedure was most often performed for pancreatic ductal adenocarcinoma at JHH/UPMC (88% vs 87% vs 97%), whereas patients in Europe had larger tumors (median 40 vs 30 vs 34 mm). Patients in Europe and at WMUH were more likely to have a pathologic American Joint Committee on Cancer (AJCC) T stage 3 cancer or higher (90% vs 98% vs 74%) than patients at JHH/UPMC.

**Table 1:** Patient characteristics per cohort

Baseline	Design cohort		Validation cohort		*P* Value
	*n* = 71	%	*n* = 120	%	
Female sex	34	48	53	44	0.654
Median age: years (IQR)	60 (52–67)		64 (58–71)		0.009
Mean age (years)	59 ± 10.6		63 ± 10.0		
Median BMI: kg/m^2^ (IQR)	24.0 (24–26.3)		24.4 (21.8–27.2)		0.353
Mean BMI (kg/m^2^)	24.3 ± 3.6		24.7 ± 4.2		
ASA					< 0.001
ASA 1	12	17	2	2	
ASA 2	53	75	50	42	
ASA 3 or 4	6	8	68	57	
Abdominal surgery history ≥ 1	22	31	53	44	0.061
Neoadjuvant therapy					< 0.001
None	35	49	28	23	
Chemotherapy	16	23	33	28	
Radiotherapy	1	1	2	2	
Both or chemoradiation	19	27	57	48	
Hepatic artery embolization	16	23	46	38	0.037
Left gastric artery embolization	6	8	19	16	0.185
Tumor characteristics (pathology)					
Ductal adenocarcinoma	62	87	113	94	0.194
Median tumor size: mm (IQR)	40 (34–50)		33 (22–45)		< 0.001
Mean tumor size (mm)	47 ± 29		34 ± 18		
AJCC^a^					
T stage ≥ 3	64	90	101	84	0.046
N stage > 0	46	66	64	53	0.168
M stage > 0	1	2	4	3	0.655

IQR, interquartile range; BMI, body mass index; ASA, American Society of Anesthesiologists; AJCC, American Joint Committee on Cancer

^a^Based on the 7th AJCC criteria^23^

### Perioperative and Long-Term Outcomes

Outcomes are presented in Table 2. Standard DP-CAR (i.e., without venous resection or arterial reconstruction) was most often performed at JHH/UPMC (Europe vs WMUH vs JHH/UPMC: 73% vs 38% vs 84%, respectively), and left gastric artery-sparing DP-CAR was often performed at WMUH (46%), but never in Europe or at JHH/UPMC. The rate of minimally invasive surgery was 18 (26%) at JHH/UPMC and negligible in Europe and at WMUH. The rates for multivisceral resection were comparable between Europe and WMUH (42% vs 42%) but lower at JHH/UPMC (30%).

**Table 2:** Outcomes per cohort

Perioperative	Design cohort		Validation cohort		*P* Value
	*n* = 71	%	*n* = 120	%	
Treated at high-volume DP-CAR centera^a^	8	11	120	100	< 0.001
Minimally invasive approach	2	3	18	15	0.012
Median operative time: min (IQR)	343 (248–425)		350 (291–447)		0.103
Mean operative time (min)	346 ± 122		380 ± 131		
Additional organs resected^b^					
None	41	58	78	65	0.361
Stomach	9	13	8	7	0.190
Liver	3	4	3	3	0.672
Kidney	3	4	3	3	0.672
Adrenal gland	17	24	31	26	0.864
DP-CAR type					< 0.001
Standard DP-CAR	52	73	79	66	
SMV/portal vein resection^c^	10	14	14	12	
Hepatic artery reconstruction	9	13	5	4	
Left gastric artery preservation/reconstruction	0	–	23	19	
Median EBL: mL (IQR)	560 (350–1450)		560 (300–1100)		0.374
Mean EBL (mL)	1015 ± 1145		996 ± 1502		
Blood transfusion for bleeding (< 72 h)	22	33	17	14	0.005
Residual status overall					0.206
R0 (> 1-mm margin)	38	55	75	63	
R1 (< 1-mm margin)	29	42	38	32	
R2 (macroscopically positive)	2	3	1	1	
90-Day outcomes					
Mortality	11	16	7	6	0.077
Clavien-Dindo 3a–4b complication	18	25	33	28	0.866
Post-pancreatectomy hemorrhage^d^	6	8	9	8	0.787
Liver ischemia/infarction	12	19	28	23	0.575
Gastric ischemia	5	7	13	11	0.452
Abdominal cavity infection	4	6	23	19	0.016
Pancreatic fistula grade B/C^d^	15	21	27	23	0.858
Delayed gastric emptying grade B/C^d^	11	15	12	10	0.495
Reoperation	10	14	6	5	0.018
Median hospital stay: days (IQR)	17 (11–26)		11 (7–21)		0.005
Mean hospital stay (days)	20 ± 14		18 ± 21		
Unplanned readmission	9	13	38	32	0.005
Long-term outcomes					
Adjuvant treatment					0.261
None	23	32	26	22	
Chemotherapy	41	58	72	60	
Radiotherapy	2	3	3	3	
Both or chemoradiation	2	3	10	8	
Unknown	3	4	10	8	
Median follow-up: days (IQR)	309 (128–617)		447 (207–939)		0.019
Median overall survival: months (95% CI)	20 (10–36)		21 (16–26)		

IQR, interquartile range; SMV, superior mesenteric vein; EBL, estimated blood loss; AJCC, American Joint Committee on Cancer; ASA, American Society of Anesthesiologists; CI, confidence interval

^a^Mean volume of 1 per year between 1 January 2014 and 31 December 2016

^b^Other than celiac axis, gallbladder, pancreas, or spleen

^c^Excluding side bite

^d^International Study Group on Pancreatic Surgery (ISGPS) definition^16^^–^18

The 90-day mortality rate was the highest in Europe (16% vs 8% vs 4%), and major morbidity was highest at WMUH (25% vs 36% vs 21%). Gastric ischemia was observed in similar proportions (7% vs 10% vs 11%). Strikingly, no liver ischemia was observed at JHH/UPMC (19% vs 56% vs 0%). The JHH/UPMC cohort had the shortest hospital stay (17 vs 21 vs 8 days) but the highest readmission rate (13% vs 14% vs 44%). The WMUH cohort had the highest rate of adjuvant therapy (63% vs 80% vs 74%), but the median overall survival, including that after non-pancreatic ductal adenocarcinoma, was highest for the JHH/UPMC cohort (20 vs 16 vs 24 months).

### 90-Day Mortality Risk Score

Univariate screening based on baseline characteristics (Supplement 2A) did not show any new predictors for 90-day mortality. Notably, preoperative embolization was not associated with lower rates of 90-day mortality. Screening based on perioperative factors (Supplement 2B) showed that patients without 90-day mortality were significantly more likely to be treated at a high-volume DP-CAR center (70% vs 39%; *P* = 0.015) and to have lower operative blood loss (median 500 vs 1050 mL; *P* = 0.021). The observed 90-day mortality rate was 5.5% (95% CI 1.5–9.5%) at 5 high-volume DP-CAR centers (2 US, 1 Japanese, and 2 European centers) and 18% (95% CI 7.9–28%) at 18 low-volume DP-CAR centers (*P* = 0.015). Notably, the type of DP-CAR was not associated with 90-day mortality (*P* = 0.458).

**Fig. 2:**
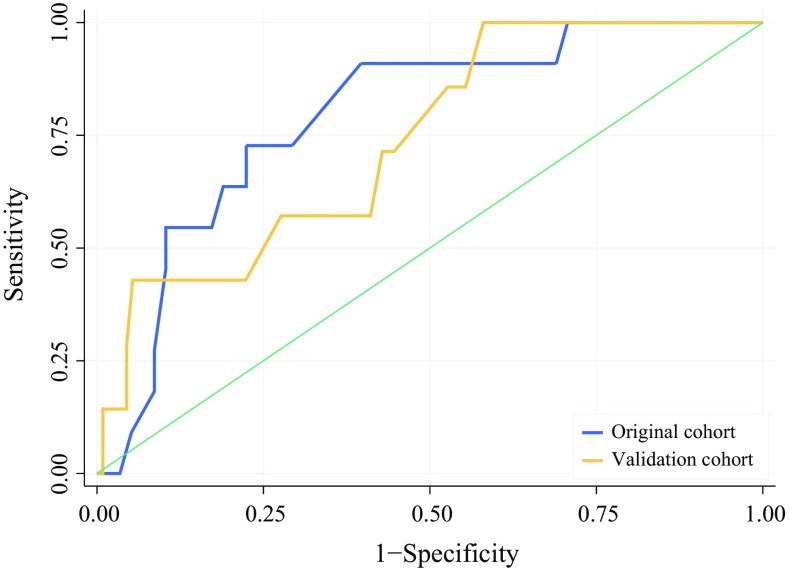
Discrimination curves for 90-day mortality prediction. Receiver operator curves (ROC) for the 90-day mortality prediction model. The area under the curve (AUC) was 0.79 (95% confidence interval [CI], 0.65–0.93) in the design cohort (*n* = 71) and 0.74 (95% CI, 0.56–0.92) in the validation cohort (*n* = 120). The difference is not significant (*P* = 0.642)

The original risk score performance for distal pancreatectomies is presented in Supplement 3. At model application in both DP-CAR cohorts, the discriminatory power remained stable between the design and validation cohorts (AUC 0.79 vs 0.74; *P* = 0.642; Fig. 2). After baseline adjustment for outcome incidence, calibration was inadequate in the validation cohort (Supplement 4A). We improved model calibration by including low- versus high-volume DP-CAR center as a covariate in the prediction model (Supplement 4B), based on an odds ratio (OR) of 3.71. This translated to 6.5 points on the risk score scale (Table 3).

**Table 3:** Risk score adjustment^a^

Risk factor	Original *β* coefficient	Original score	Adjusted *β* coefficient	Adjusted score
Age per 10 years (40–90)	0.11	0.5	–	0.5
Female with ASA 1–2	0	0	–	0
Female with ASA 3–4	0.52	2.5	–	2.5
Male (any ASA)	0.88	4.5	–	4.5
BMI per 10 points (20–50)	0.39	2	–	2
Multivisceral resection^a^	0.42	2	–	2
Open surgery	0.49	2.5	–	2.5
High-volume DP-CAR center^b^	–	–	− 1.31	− 6.5
Intercept	− 2.82	–	− 3.96	–
Total		6–23.5		0–23.5

Model coefficients were obtained from the original design dataset^7^ to determine the risk score by rounding and multiplication. The intercept was adjusted for the risk of 90-day mortality in the design cohort (*n* = 71); a coefficient for high-volume center was added

ASA, American Society of Anesthesiologists; BMI, body mass index; DP-CAR, distal pancreatectomy with celiac axis resection

^a^Definition: other than celiac axis, gallbladder, pancreas, or spleen

^b^Annual volume ≥ 1 DP-CAR

Final model discrimination, after addition of the volume covariate, remained good (AUC 0.79; 95% CI, 0.68–0.90). A clinical risk calculator is accessible online at www.pancreascalculator.com.

## Discussion

In this international study of 191 patients treated at 23 centers, DP-CAR was associated with a 90-day mortality rate of 5.5% in 5 high-volume DP-CAR centers (≥ 1 DP-CAR/year) and 18% in 18 low-volume DP-CAR centers and an additional major morbidity rate of 27%. For 174 patients with pancreatic ductal adenocarcinoma, the R0-resection rate was 60%, and the median overall survival period was 19 months.

We created and validated a clinical risk score for 90-day mortality to improve patient selection. The discriminatory power of the risk score was similar between the design and validation cohorts (AUC 0.79 vs 0.74; *P* = 0.642). Low annual DP-CAR volume (< 1) was the strongest predictor (OR 3.71) of 90-day mortality. The risk score included age, sex, BMI, ASA classification, multivisceral surgery, open versus minimally invasive surgery, and low- versus high-volume DP-CAR center.

In a recent systematic review of DP-CAR, the 90-day mortality rate among 113 patients was 3.5%.^2^ In addition, two single-center studies reported 90-day mortality rates of 18% and 17%, respectively.^8^,^9^ The overall 90-day mortality rate in the current international multicenter study among 191 patients was 9.5%. Therefore, it seems likely that publication bias affected prior estimates of the 90-day mortality rate in the systematic review.

On the other hand, our study confirmed previous findings on the volume-outcome relationship in pancreas surgery. For example, a nationwide registry study^25^ found a significantly increased risk of 90-day mortality after pancreatoduodenectomy (OR 2.59; 95% CI 1.32–5.09) in the lowest- versus the highest-volume centers (cutoff, 40 pancreatoduodenectomies per year). Similar volume-outcome associations for 90-day mortality have been found by others.^22^,^26^

We noted a few interesting variations in clinical strategies between the three very-high-volume centers in the validation cohort. First, prolonged neoadjuvant treatment is routinely applied (FOLFIRINOX or nab-paclitaxel plus gemcitabine, up to 8 cycles) at JHH and UPMC to assess both tumor biology (aggressiveness) and patient fitness. Second, although some multivisceral resections were necessary during the operation, preoperatively apparent tumor involvement of additional organs is an absolute contraindication for surgery at all three centers. Third, all three centers recognize the need to preserve as much organ perfusion as possible. At WMUH, this is addressed by routine performance of preoperative left gastric and hepatic artery embolization or by preservation or reconstruction of the left gastric artery (middle colic artery bypass). At JHH and UPMC, surgeons refrain from resecting the superior mesenteric artery and perform only partial portal vein resection (side-bites/wedge resection) when involvement is detected intraoperatively. This could explain the low liver ischemia rates and superior survival at UPMC/JHH. Unfortunately, the subgroups in this study remained too small to study the effect of these strategies on 90-day mortality.

In addition to the clinical strategies described earlier, outcomes such as 90-day mortality after DP-CAR may be improved by using the proposed clinical risk score. Avoiding DP-CAR for high-risk patients would be an obvious step to lowering mortality. Given the limited survival benefit after resection of pancreatic ductal adenocarcinoma, a mortality risk exceeding 10–20% would not seem justified. Alternatively, the clinical risk score should be used for baseline risk adjustment in future studies comparing specific techniques or center performance.

Despite these practical applications, continuous model reevaluation is important to maintain accuracy. For example, further centralization of pancreatic surgery may shift the definition of “high-volume”. Notably, the high-volume centers in this study all performed more than 90 (median 175; interquartile range [IQR], 105–293) pancreatic resections per year.

This study had some limitations. First, despite the large cohorts, it lacked sufficient power to detect less obvious predictors of 90-day mortality. As a result, we may have missed predictors in our clinical risk score. Second, to increase power, all DP-CAR variations were grouped together, whereas outcomes in fact may differ. Future studies should aim to study the differences in long-term outcomes between DP-CAR variations. Third, surgeon and center experience are perhaps the most important determinant of a successful outcome after surgery. Although this is partly reflected in the risk score, surgeons should always consider their individual training and experience. Fourth, we used annual case volume as a surrogate marker for the experience of the surgical team. We acknowledge that this may not capture the full complexity of factors that contribute to improved outcomes at these centers. Moreover, referral patterns may shift and/or external expertise may be acquired. Therefore, the high-volume threshold presented in this study should be used only as a starting point for further discussion on centralization. Fifth, median overall survival times should be interpreted with caution because the median follow-up time was 385 days in both cohorts combined. Sixth, there was a considerable time differential (4–7 years) between the design and validation cohorts, which may have introduced a time-dependent bias. Although this may be the case for the effect of chemo-/radiotherapy regimens on survival, we previously ruled out any differences over time for morbidity and mortality outcomes in the European population.^7^ Finally, although arguably part of the most important outcomes in oncologic surgery, quality-of-life measures were not available for this retrospective study.

This study has some important practical implications and offers important starting points for further study. First, although the consensus that rare and high-risk procedures such as DP-CAR should be limited to very-high-volume centers is well established, optimal volume thresholds for this procedure have not been determined to date. Even a selective approach (e.g., favorable patient risk factors) to DP-CAR should be avoided at low-volume centers because this would decrease the annual DP-CAR volume even further.

Second, important practice variations between high-volume DP-CAR centers, such as the application of preoperative embolization or chemoradiotherapy, were noted. Although these variations did not modify the effect of volume on 90-day mortality in this study, they may indeed have an impact on long-term outcomes such as survival. These effects are likely to be more evident in a broader study setting such as in the Arterial Study Network.^27^

Third, the actual impact of improved patient selection on outcomes after DP-CAR needs further study.

In conclusion, this study presents the largest international series on DP-CAR to date and includes a validated clinical risk score for 90-day mortality. The main finding is that annual DP-CAR case volume is the most important predictor for 90-day mortality. Future studies should aim at (prospectively) validating the clinical risk score, which was made available online at www.pancreascalculator.com.

## Electronic supplementary material

Below is the link to the electronic supplementary material.

Supplementary material 1 (DOCX 435 kb)
