# Supplementary material for: Outcomes and Risk Score for Distal Pancreatectomy with Celiac Axis Resection (DP-CAR): An International Multicenter Analysis
Source: Ann Surg Oncol. 2019 Jan 4;26(3):772–81. doi: 10.1245/s10434-018-07101-0 (PMC6373251; doi:10.1245/s10434-018-07101-0)
Supplement: Supplementary file 1 — Supplementary material 1 (DOCX 435 kb) [file 10434_2018_7101_MOESM1_ESM.docx]

SUPPLEMENT 1A. BASELINE CHARACTERISTICS PER REGION/CENTER

|  | Design cohort | | Validation cohort | | | |
| --- | --- | --- | --- | --- | --- | --- |
|  | Europe |  | WMUH | | JHH/UPMC | |
| **Baseline** | n=71 |  | n=50 |  | n=70 |  |
| Female sex, No. (%) | 34 | (48) | 18 | (36) | 35 | (50) |
| Age, median (IQR), y | 60 | (52-67) | 68 | (62-72) | 62 | (56-68) |
| Mean (SD), y | 59 | (10.6) | 66 | (8) | 61 | (11) |
| Body-mass-index, median (IQR), kg/m2 | 24.0 | (24-26.3) | 22.1 | (19.9-24.4) | 26.0 | (23.8-28.9 |
| Mean (SD), kg/m2 | 24.3 | (3.6) | 22.1 | (3.2) | 26.6 | (3.9) |
| ASA-classification, No. (%) |  |  |  |  |  |  |
| ASA-1 | 12 | (17) | 2 | (4) | 0 | (0) |
| ASA-2 | 53 | (75) | 39 | (78) | 11 | (16) |
| ASA-3 or ASA-4 | 6 | (8) | 9 | (19) | 59 | (84) |
| Abdominal surgery history ≥1, No. (%) | 22 | (31) | 21 | (42) | 32 | (46) |
| Neoadjuvant therapy, No. (%) |  |  |  |  |  |  |
| None | 35 | (49) | 24 | (48) | 4 | (6) |
| Chemotherapy | 16 | (23) | 14 | (28) | 19 | (27) |
| Radiotherapy | 1 | (1) | 0 | (-) | 2 | (3) |
| Both or chemoradiation | 19 | (27) | 12 | (24) | 45 | (64) |
| Hepatic artery embolization, No. (%) | 16 | (23) | 46 | (92) | 0 | (-) |
| Left gastric artery embolization, No. (%) | 6 | (8) | 19 | (38) | 0 | (-) |
| **Tumor characteristics (pathology)** |  |  |  |  |  |  |
| Ductal adenocarcinoma, No. (%) | 62 | (87) | 44 | (88) | 68 | (97) |
| Tumor size, median (IQR), mm | 40 | (34-50) | 30 | (23-40) | 34 | (22-45) |
| Mean (SD), mm | 47 | (29) | 34 | (18) | 35 | (18) |
| AJCC Staging*, No. (%) |  |  |  |  |  |  |
| T-stage ≥ 3 | 64 | (90) | 49 | (98) | 52 | (74) |
| N-stage > 0 | 46 | (66) | 31 | (62) | 33 | (47) |
| M-stage > 0 | 1 | (2) | 1 | (2) | 3 | (4) |

* Based on the 7^th^ AJCC criteria.^24^

Abbreviations: ASA, American Society of Anesthesiologists.

SUPPLEMENT 1B. OUTCOMES PER REGION/CENTER

|  | Design cohort | | Validation cohort | | | |
| --- | --- | --- | --- | --- | --- | --- |
|  | Europe |  | WMUH |  | JHH/UPMC | |
| **Perioperative** | n=71 |  | n=50 |  | n=70 |  |
| Treated at DP-CAR high-volume center*, No. (%) | 8 | (11) | 50 | (100) | 70 | (100.0) |
| Minimally invasive approach, No. (%) | 2 | (3) | 0 | (-) | 18 | (26) |
| Operative time, median (IQR), min. | 343 | (248-425) | 342 | (283-406) | 368 | (298-480) |
| Mean (SD), min. | 346 | (122) | 360 | (119) | 398 | (141) |
| Additional organs resected**, No. (%) |  |  |  |  |  |  |
| None | 41 | (58) | 28 | (56) | 49 | (70) |
| Stomach | 9 | (13) | 1 | (2) | 7 | (10) |
| Liver | 3 | (4) | 0 | (-) | 3 | (4) |
| Kidney | 3 | (4) | 1 | (2) | 2 | (3) |
| Adrenal gland | 17 | (24) | 22 | (44) | 9 | (13) |
| DP-CAR variation, No. (%) |  |  |  |  |  |  |
| Standard DP-CAR | 51 | (72) | 19 | (38) | 59 | (84) |
| SMV/Portal vein resection *** | 10 | (14) | 8 | (16) | 6 | (9) |
| Superior mesenteric artery resection | 1 | (1) | 0 | (-) | 0 | (-) |
| Hepatic artery reconstruction | 9 | (13) | 0 | (-) | 5 | (7) |
| Left gastric artery preservation/reconstruction | 0 | (-) | 23 | (46) | 0 | (-) |
| Estimated blood loss, median (IQR), mL | 560 | (350-1450) | 500 | (190-1165) | 750 | (300-1000) |
| Mean (SD), mL | 1015 | (1145) | 1054 | (1914) | 937 | (955) |
| Blood transfusion for bleeding (<72 hrs), No. (%) | 22 | (33) | 10 | (20.0) | N/A | N/A |
| Residual status overall, No. (%) |  |  |  |  |  |  |
| R0 (>1 mm margin) | 38 | (55) | 31 | (62) | 44 | (63) |
| R1 (<1 mm margin) | 29 | (42) | 18 | (36) | 20 | (29) |
| R2 (macroscopically positive) | 2 | (3) | 1 | (2) | 0 | (-) |
| **90-day outcomes** |  |  |  |  |  |  |
| Mortality (Clavien-Dindo 5), No. (%) | 11 | (16) | 4 | (8) | 3 | (4) |
| Clavien-Dindo 3a-4b complication | 18 | (25) | 18 | (36) | 15 | (21) |
| Post-pancreatectomy hemorrhage****, No. (%) | 6 | (8) | 7 | (14) | 0 | (-) |
| Liver ischemia/infarction, No. (%) | 12 | (19) | 28 | (56) | 0 | (-) |
| Gastric ischemia, No. (%) | 5 | (7) | 5 | (10) | 8 | (11) |
| Abdominal cavity infection, No. (%) | 4 | (6) | 17 | (34) | 6 | (9) |
| Pancreatic fistula grade B/C****, No. (%) | 15 | (21) | 13 | (26) | 14 | (20) |
| Delayed gastric emptying grade B/C****, No. (%) | 11 | (15) | 9 | (18) | 3 | (4) |
| Reoperation, No. (%) | 10 | (14) | 4 | (8) | 2 | (3) |
| Length of hospital say, median (IQR), days | 17 | (11-26) | 21 | (15-33) | 8 | (7-10) |
| Mean (SD), days | 20 | (14) | 30 | (28) | 10 | (6) |
| Unplanned readmission, No. (%) | 9 | (13) | 7 | (14) | 31 | (44) |
| **Long-term outcomes** |  |  |  |  |  |  |
| Adjuvant treatment, No. (%) |  |  |  |  |  |  |
| None | 23 | (32) | 10 | (20) | 18 | (26) |
| Chemotherapy | 41 | (58) | 40 | (80) | 32 | (46) |
| Radiotherapy | 2 | (3) | 0 | (0) | 3 | (4) |
| Both or chemoradiation | 2 | (3) | 0 | (0) | 10 | (14) |
| Unknown | 3 | (4) | 0 | (0) | 7 | (10) |
| Follow-up time, median (IQR), d | 309 | (128-617) | 466 | (240-952) | 435 | (197-791) |
| Overall survival, median (95% CI), d | 617 | (317-1108) | 500 | (384-761) | 723 | (537-1044) |

* Mean volume of 1 per year between January 1^st^ 2014 and December 31^st^ 2016

** Other than celiac axis, gallbladder, pancreas, or spleen.

*** Excluding side-bite

**** ISGPS definition^15–17^

SUPPLEMENT 2A. UNIVARIABLE SCREEN FOR 90-DAY MORTALITY

|  | 90-day mortality | | No 90-day mortality | |  |
| --- | --- | --- | --- | --- | --- |
| **Baseline** | n=18 |  | n=171 |  | P-value |
| Female sex, No. (%) | 3 | (17) | 84 | (49) | 0.011 |
| Age, median (IQR), y | 66 | (56-70) | 63 | (56-69) | 0.532 |
| Mean (SD), y | 63 | (11) | 62 | (10) |  |
| Body-mass-index, median (IQR), kg/m2 | 25.2 | (22-27) | (24.1) | (22-27) | 0.488 |
| Mean (SD), kg/m2 | 24.8 | (3.3) | (24.5) | (4.1) |  |
| ASA-classification, No. (%) |  |  |  |  | 0.530 |
| ASA-1 | 0 | (-) | 14 | (8) |  |
| ASA-2 | 12 | (67) | 90 | (53) |  |
| ASA-3 or ASA-4 | 6 | (33) | 67 | (39) |  |
| Abdominal surgery history ≥1, No. (%) | 8 | (44) | 65 | (41) | 0.805 |
| Neoadjuvant therapy, No. (%) |  |  |  |  | 0.122 |
| None | 9 | (50) | 54 | (32) |  |
| Chemotherapy | 1 | (6) | 48 | (28) |  |
| Radiotherapy | 0 | (-) | 3 | (2) |  |
| Both or chemoradiation | 8 | (44) | 66 | (39) |  |
| Hepatic artery embolization, No. (%) | 8 | (44) | 54 | (32) | 0.297 |
| Left gastric artery embolization, No. (%) | 1 | (6) | 24 | (14) | 0.476 |
| **Tumor characteristics (pathology)** |  |  |  |  |  |
| Ductal adenocarcinoma, No. (%) | 16 | (89) | 156 | (91) | 0.346 |
| Tumor size, median (IQR), mm | 40 | (25-45) | 35 | (25-45) | 0.533 |
| Mean (SD), mm | 40 | (20) | 39 | (24) |  |
| AJCC Staging*, No. (%) |  |  |  |  |  |
| T-stage ≥ 3 | 17 | (94) | 146 | (87) | 0.879 |
| N-stage > 0 | 15 | (82) | 95 | (57) | 0.041 |
| M-stage > 0 | 0 | (-) | 5 | (3) | >0.99 |
| Risk score, median (IQR) | 20.0 | (16-23) | 15 | (12.5-16.5) | <0.001 |

* Based on the 7^th^ AJCC criteria.^24^

Abbreviations: AJCC, American Joint Committee on Cancer; ASA, American Society of Anesthesiologists.

SUPPLEMENT 2B. UNIVARIABLE SCREEN FOR 90-DAY MORTALITY

|  | 90-day mortality | | No 90-day mortality | |  |
| --- | --- | --- | --- | --- | --- |
| **Perioperative** | n=18 |  | n=171 |  | P-value |
| Treated at DP-CAR high-volume center*, No. (%) | 7 | (39) | 120 | (70) | 0.015 |
| Minimally invasive approach, No. (%) | 0 | (-) | 20 | (12) | 0.119 |
| Operative time, median (IQR), min. | 305 | (270-427) | 346 | (283-430) | 0.579 |
| Mean (SD), min. | 383 | (191) | 364 | (120) |  |
| Additional organs resected** No. (%) |  |  |  |  |  |
| None | 10 | (56) | 107 | (63) | 0.614 |
| Gallbladder | 2 | (11) | 37 | (22) | 0.374 |
| Stomach | 3 | (17) | 14 | (8) | 0.210 |
| Liver | 0 | (-) | 6 | (4) | >0.99 |
| Kidney | 2 | (11) | 4 | (2) | 0.102 |
| Adrenal gland | 7 | (39) | 40 | (23) | 0.159 |
| DP-CAR variation, No. (%) |  |  |  |  | 0.458 |
| Standard DP-CAR | 15 | (83) | 113 | (66) |  |
| SMV/Portal vein resection *** | 2 | (11) | 21 | (12) |  |
| Superior mesenteric artery resection | 0 | (-) | 1 | (1) |  |
| Hepatic artery reconstruction | 1 | (6) | 13 | (8) |  |
| Left gastric artery preservation/reconstruction | 0 | (-) | 23 | (13) |  |
| Estimated blood loss, median (IQR), mL | 1050 | (563-1582) | 500 | (300-1135) | 0.021 |
| Mean (SD), mL | 2074 | (3058) | 883 | (1005) |  |
| Blood transfusion for bleeding (<72 hrs), No. (%) | 9 | (56) | 29 | (18) | 0.001 |
| Residual status overall, No. (%) |  |  |  |  | 0.005 |
| R0 (>1 mm margin) | 6 | (35) | 105 | (64) |  |
| R1 (<1 mm margin) | 9 | (53) | 58 | (35) |  |
| R2 (macroscopically positive) | 2 | (12) | 1 | (1) |  |
| **Postoperative (90 days)** |  |  |  |  |  |
| Post-pancreatectomy hemorrhage****, No. (%) | 8 | (44) | 7 | (4) | <0.001 |
| Liver ischemia/infarction, No. (%) | 9 | (50) | 31 | (19) | 0.005 |
| Gastric ischemia, No. (%) | 7 | (39) | 10 | (6) | <0.001 |
| Abdominal cavity infection, No. (%) | 6 | (35) | 21 | (12) | 0.021 |
| Pancreatic fistula grade B/C****, No. (%) | 6 | (33) | 36 | (21) | 0.247 |
| Delayed gastric emptying grade B/C****, No. (%) | 9 | (50) | 14 | (9) | <0.001 |
| Length of hospital say, median (IQR), days | 24 | (12-40) | 13 | (8-21) | 0.001 |
| Mean (SD), days | 27 | (18) | 18 | (19) |  |

2 Patients were loss to follow-up before 90-days.

* Mean volume of 1 per year between January 1st 2014 and December 31st 2016

** Other than celiac axis, gallbladder, pancreas, or spleen.

*** Excluding side-bite

**** ISGPS definition^15–17^

SUPPLEMENT 3. RISK SCORE PERFORMANCE IN THE DESIGN DATABASE (N=1661)

The original design database contained 1661 distal pancreatectomy cases without arterial resection and comprised the 2014 pancreas-targeted ACS-NSQIP® (<https://www.facs.org/quality-programs/acs-nsqip>), an institutional database at BIDMC (2006-2015), and data from a mandatory registry among 17 Dutch Pancreatic Cancer Group centers (2005-2015). Inclusion criteria were: age 18 or over, surgical indication for solid tumors or cysts, open-, laparoscopic-, or robot-assisted resection. Patients were excluded in case of chronic pancreatitis as only diagnosis, arterial resection, or unrelated multivisceral resection. The primary outcome was composite major morbidity, a composite 30-day outcome comprising death, reoperation related to the index procedure, pancreatic fistula requiring non-surgical or surgical re-intervention, postoperative organ space surgical site infection, pulmonary embolism, stroke or cerebrovascular accident, myocardial infarction or cardiac arrest requiring cardiopulmonary resuscitation, septic shock, or length of initial hospital stay above the 90th percentile of the appropriate population (≥10 days in NSQIP® and BIDMC; ≥16 days in DPCG).

A prediction model for composite major morbidity was designed using purposeful selection (including a univariate screen) with logistic regression. The model was designed in NSQIP® and cross-validated consecutively using the BIDMC and DPCG data. Validated risk factors were minimally-invasive surgery (OR 0.61, CI 0.44-0.79), age (per decade; OR 1.12, CI 1.00-1.24), body mass index (per increment of 10; OR 1.48, CI 1.18-1.90), male sex (OR 2.41, CI 1.60-3.33), females with American Society of Anesthesiologists classification 3 or 4 (OR 1.69, CI 1.07-2.41), and intraductal papillary mucinous neoplasm (OR 0.61, CI 0.37-0.96). Multivisceral resection (OR 1.52, CI 0.99-2.46) was included because it’s know association with postoperative morbidity. Below, we present the model performance with outcomes 30-day composite major morbidity (Figure 3A, 3B) and 30-day mortality (Figure 3C, 3D).

3A 3B

3C 3D

CAPTION: Validation of the risk prediction model in 1661 distal pancreatectomy patients from the design dataset. A. Receiver-operator-curves (ROC) for major morbidity (AUC 0.66, CI: 0.62-0.69); B. Calibration curve for major morbidity; C. Receiver-operator-curves (ROC) for mortality (AUC 0.71, CI: 0.63-0.80); D. Calibration curve for mortality.

SUPPLEMENT 4. CALIBRATION PLOT FOR 90-DAY MORTALITY PREDICTION

CAPTION: Calibration plots for the predicted probability of 90-day mortality: (A) applying only baseline risk adjustment and (B) including a predictor for high versus low yearly DP-CAR volume. The dotted 45° lines denote a hypothetical perfect agreement between predicted probability and observed incidence per risk quartile. The smoothed lines approximate the actual agreement observed in both cohorts.
